# Bi‐allelic *KARS1* pathogenic variants affecting functions of cytosolic and mitochondrial isoforms are associated with a progressive and multisystem disease

**DOI:** 10.1002/humu.24210

**Published:** 2021-05-11

**Authors:** Gerarda Cappuccio, Camilla Ceccatelli Berti, Enrico Baruffini, Jennifer Sullivan, Vandana Shashi, Tamison Jewett, Tara Stamper, Silvia Maitz, Francesco Canonico, Anya Revah‐Politi, Gabriel S. Kupchik, Kwame Anyane‐Yeboa, Vimla Aggarwal, Andreas Benneche, Eirik Bratland, Siren Berland, Felice D'Arco, Cesar A. Alves, Adeline Vanderver, Daniela Longo, Enrico Bertini, Annalaura Torella, Vincenzo Nigro, Telethon Undiagnosed Diseases Program, Alessandra D'Amico, Marjo S. van der Knaap, Paola Goffrini, Nicola Brunetti‐Pierri

**Affiliations:** ^1^ Department of Translational Medicine Federico II University Naples Italy; ^2^ Telethon Institute of Genetics and Medicine Pozzuoli Naples Italy; ^3^ Department of Chemistry, Life Sciences and Environmental Sustainability University of Parma Parma Italy; ^4^ Division of Medical Genetics, Department of Pediatrics Duke University Medical Center Durham North Carolina USA; ^5^ Department of Pediatrics, Section on Medical Genetics Wake Forest University School of Medicine, Winston‐Salem North Carolina USA; ^6^ Clinical Pediatric Genetics Unit, Pediatrics Clinics, MBBM Foundation, Hospital San Gerardo Monza Italy; ^7^ Department of Neuroradiology, San Gerardo Hospital, ASST di Monza Università degli Studi di Milano Bicocca Monza Italy; ^8^ Department of Pathology and Cell Biology, Institute for Genomic Medicine Columbia University Irving Medical Center New York New York USA; ^9^ Division of Medical Genetics, Maimonides Children's Hospital of Brooklyn at Maimonides Medical Center, Downstate Medical Center State University of New York New York New York USA; ^10^ Department of Pediatrics Institute for Genomic Medicine Columbia University Medical Center New York New York USA; ^11^ Department of Pathology and Cell Biology Columbia University Irving Medical Center New York New York USA; ^12^ Department of Medical Genetics Haukeland University Hospital Bergen Norway; ^13^ Department of Paediatric Neuroradiology Great Ormond Street Hospital for Children NHS Foundation Trust London UK; ^14^ Division of Neuroradiology, Department of Radiology Children's Hospital of Philadelphia Philadelphia Pennsylvania USA; ^15^ Division of Neurology, Children's Hospital of Philadelphia and Perelman School of Medicine University of Pennsylvania Philadelphia Pennsylvania USA; ^16^ Department of Diagnostic Imaging Pediatric Hospital Bambino Gesù Rome Italy; ^17^ Department of Neuroscience Unit of Neuromuscular and Neurodegenerative Diseases, IRCCS Bambino Gesù Children's Hospital Rome Italy; ^18^ Department of Precision Medicine University of Campania Luigi Vanvitelli Naples Italy; ^19^ Department of Advanced Biomedical Sciences Federico II University Naples Italy; ^20^ Department of Child Neurology, Amsterdam Leukodystrophy Center, Emma Children's Hospital Amsterdam University Medical Centers and Amsterdam Neuroscience Amsterdam The Netherlands; ^21^ Department of Functional Genomics, Center for Neurogenomics and Cognitive Research VU University Amsterdam The Netherlands

**Keywords:** *KARS*, *KARS1*, LysRS, lysyl‐transfer RNA synthetase, mitochondrial disease

## Abstract

*KARS1* encodes a lysyl‐transfer RNA synthetase (LysRS) that links lysine to its cognate transfer RNA. Two different *KARS1* isoforms exert functional effects in cytosol and mitochondria. Bi‐allelic pathogenic variants in *KARS1* have been associated to sensorineural hearing and visual loss, neuropathy, seizures, and leukodystrophy. We report the clinical, biochemical, and neuroradiological features of nine individuals with *KARS1*‐related disorder carrying 12 different variants with nine of them being novel. The consequences of these variants on the cytosol and/or mitochondrial LysRS were functionally validated in yeast mutants. Most cases presented with severe neurological features including congenital and progressive microcephaly, seizures, developmental delay/intellectual disability, and cerebral atrophy. Oculo‐motor dysfunction and immuno‐hematological problems were present in six and three cases, respectively. A yeast growth defect of variable severity was detected for most variants on both cytosolic and mitochondrial isoforms. The detrimental effects of two variants on yeast growth were partially rescued by lysine supplementation. Congenital progressive microcephaly, oculo‐motor dysfunction, and immuno‐hematological problems are emerging phenotypes in *KARS1*‐related disorder. The data in yeast emphasize the role of both mitochondrial and cytosolic isoforms in the pathogenesis of *KARS1*‐related disorder and supports the therapeutic potential of lysine supplementation at least in a subset of patients.

## 1 INTRODUCTION

Aminoacyl‐transfer RNA synthetases (ARSes) are involved in the translation of messenger RNA (mRNA) into proteins. Each of the 37 known ARSes binds a specific amino acid to its corresponding transfer RNA (tRNA). Lysyl‐transfer RNA synthetase (LysRS) encoded by *KARS1* loads lysine to its cognate tRNA. In contrast to most ARSes, cytosolic and mitochondrial LysRS are not encoded by two separate genes but are generated by alternative splicing of one *KARS1* gene. The cytosolic isoform of LysRS is generated by pre‐mRNA splicing of exons 1–3 (*cytKARS1*, NM_005548.2), whereas the mitochondrial isoform is generated by a pre‐mRNA that contains exon 2 encoding for the mitochondrial signal peptide (*mtKARS1*, NM_001130089.1). The cytosolic isoform of LysRS is part of a multisynthetase complex (MSC) that contains nine additional tRNA synthases and three scaffold proteins (p43/AIMP1, p38/AIMP2, and p18/AIMP3) (Hei et al., 2019; Ruzzenente et al., 2018; Simos et al., 1996; Zhou et al., 2017). To date, 32 *KARS1* pathogenic variants have been reported in 43 individuals presenting with sensorineural hearing loss (Santos‐Cortez et al., 2013), optic neuropathy (Scheidecker et al., 2019), peripheral neuropathy (McLaughlin et al., 2010), hypertrophic cardiomyopathy with lactic acidosis, and Complex I and IV deficiency (Kohda et al., 2016; Verrigni et al., 2017). Few cases with more severe neurologic involvement including leukodystrophy have been grouped into a condition named congenital deafness and adult‐onset progressive leukoencephalopathy (DEAPLE, OMIM 619196) (van der Knaap et al., 2019). A similar condition with an early onset neurologic disease and spinal and brainstem calcifications named as leukoencephalopathy, progressive, infantile‐onset, with or without deafness (LEPID, OMIM 619147) has also been reported (Ardissone et al., 2018, Itoh et al., 2019, Ruzzenente et al., 2018, Zhou et al., 2017). Here, we report nine cases with severely progressive neurodegenerative and multisystem disease due to bi‐allelic *KARS1* variants that were functionally validated in yeast assays.

## 2 PATIENTS, MATERIALS, AND METHODS

### 2.1 Patients and sequencing

All cases were referred for developmental delay, intellectual disability, and/or microcephaly. The study was approved by ethic committees at Federico II University Hospital (48/16), Duke University Medical Center (00032301), and Columbia University Irving Medical Center (AAAO8410). Informed consent for either diagnostic or research investigations was obtained for all cases. Clinical, biochemical, neuroimaging, and genetic studies were available for all cases. For each case, the mitochondrial diagnostic score was attributed according to Morava et al. (2006). Genomic DNA from each proband and both parents underwent exome sequencing (ES) using site‐specific protocols and *KARS1* variants were confirmed by Sanger sequencing. ACMG classification was used for standardizing variant interpretation (Richards et al., 2015). For consistency, variant nomenclature has been provided for the longer mitochondrial isoform (NP_001123561.1) for all variants except for the variant p.(Ala2Val), that was specific for the cytosolic isoform (NP_005539.1). Predicted damaging effects of *KARS1* variants were evaluated by SIFT, PolyPhen2, Mutation Taster, PROVEAN, M‐CAP, CADD, and PhastCons. Multispecies sequence alignment of LysRS proteins was performed using CLUSTAL Omega. Amino acid residues affected by variants detected in cases were mapped on the crystal structure of the KARS‐p38 complex (PDB: 4dpg) loaded into PyMOL or RasMol.

### 2.2 Functional yeast studies

Yeast strains derived from W303‐1B (*Matα ade2‐1 leu2‐3*, *112 ura3‐1 trp1‐1 his3‐11*, and *15 can1‐100*) (Thomas & Rothstein, 1989) were grown in synthetic complete (SC) media (0.69% yeast nitrogen base without amino acids) (ForMedium) supplemented with 1 g/L drop‐out mix (DO) except amino acids and bases needed to retain plasmids and with or without lysine. Various carbon sources were added at 2% (w/v) (Carlo Erba) in the liquid phase or after solidification with 20 g/L agar (ForMedium). The 1 mg/ml 5‐fluoroorotic acid (5‐FOA) monohydrate (ForMedium) was supplemented into SC medium. YPD medium (0.5% Yeast extract, 1% Peptone, and 2% glucose) was supplemented with either 200 µg/ml geneticine disulfate or 250 µg/ml hygromycin B (Formedium). *MSK1* and *KRS1* encoding the mitochondrial and cytosolic isoforms of yeast LysRS respectively were polymerase chain reaction (PCR)‐amplified (Table S1) and cloned under their endogenous promoter into the centromeric pFL38 plasmid that includes *URA3* as selection marker. The pFL38*MSK1* and pFL38*KRS1* plasmids were separately transformed into the W303‐1B strain, and one‐step gene disruption (Wach et al., 1994) of *MSK1* or *KRS1* genes was performed in these strains because loss of *MSK1* results in instability of mitochondrial DNA (mtDNA) (Gatti & Tzagoloff, 1991), whereas loss of *KRS1* is lethal. For *MSK1* disruption, the KanMX4 cassette was amplified from the BY4741 *msk1Δ* strain. For *KRS1* disruption, the HphMX cassette flanked by *KRS1* sequences was amplified from pAG32 plasmid (Goldstein & McCusker, 1999). Transformation and selection on YPD medium supplemented with appropriate antibiotics were performed according to previous study (Gietz, 2014) to obtain W303‐1B *msk1Δ*/pFL38*MSK1* and W303‐1B *krs1Δ*/pFL38*KRS1*. Human complementary DNAs (cDNAs) encoding the *mtKARS1* (NM_001130089, purchased from OriGene) and *cytKARS1* (NM_005548 purchased from OriGene) were PCR‐amplified and cloned into the centromeric single copy expression vector pFL39‐TEToff (Nolli et al., 2015), which contains *TRP1* as selection marker and the TET‐off cassette, which is made by the *CYC1* promoter and seven repeats of the Tet operator (TetO). The *cytKARS1* cDNA was also subcloned into multicopy expression vector YEplac112‐TEToff obtained by subcloning the TET‐off promoter in YEplac112 (Gietz & Sugino, 1988). Except for the p.(Ala2Val) variant, all *KARS1* missense variants were inserted in both mitochondrial and cytosolic isoforms by PCR QuikChange™ (Agilent) using KOD Hot Start DNA Polymerase (Merck) and appropriate primers (Table S1). The p.(Ala2Val) variant was only inserted into the cytoplasmic isoform. Plasmids expressing wild‐type and mutant alleles were transformed into the corresponding deleted strain. In *msk1Δ* strains expressing the mutant alleles, the pFL38*MSK1* was lost through plasmid‐shuffling on 5‐FOA medium, as previously reported (Baruffini et al., 2010). Strains devoid of *MSK1* were maintained in YP supplemented with 2% ethanol or 2% glycerol if respiratory proficient or in SC‐W medium supplemented with 2% glucose if respiratory deficient. For oxidative growth analysis, strains were serially diluted and spotted on SC‐W agar plates, with or without lysine 50 µg/ml, supplemented with 2% glycerol or 2% glucose as control. Plates were incubated at both 28°C and 37°C. Oxygen consumption rate (OCR) was measured at 30°C from 20 mg of wet‐weight yeast cell suspensions cultured under shaking in SC medium supplemented with 0.5% glucose until exhaustion of the latter (for 18 h at 28°C or for 16 h at 37°C) measured by a Clark‐type oxygen electrode (Oxygraph System Hansatech Instruments) with 1 ml of air‐saturated respiration buffer (0.1 M phthalate–KOH, pH 5.0) and 0.5% glucose. Values for OCR were normalized to the dry weight of the cells. In *krs1Δ* strains expressing mutant *cytKARS1*, yeast viability was evaluated by analyzing growth on 5‐FOA medium supplemented with 2% glucose. For each *cytKARS1* wild‐type and mutant strain, a small patch of cells was at first inoculated in 200 µl of SC‐W‐U medium into a 96‐well microtiter plate and grown for 24 h at 28°C without shaking thereafter. Then, 2 µl of this culture was inoculated in 200 µl of SC‐W containing uracil to allow loss of the pFL38*KRS1* plasmid and grown for 30 h at 28°C without shaking. For all strains, cell concentration was between 7.5 and 8.5 OD_600_. The 5 µl of undiluted culture and 1:10 dilutions were then spotted on the appropriate 5‐FOA medium with or without lysine 50 µg/ml. Growth was assessed after incubation at 28°C or 37°C.

Western blots were performed on proteins extracted from deleted strains harboring *MSK1* or *KRS1* on pFL38 and *mtKARS1* and *cytKARS1* variants on pFL39‐TEToff and YEplac112‐TEToff, respectively. In the former case, *MSK1* gene was retained to maintain normal mtDNA levels and respiratory proficiency in all *mtKARS1* mutant strains; in the latter case, *KRS1* gene was retained to maintain the viability of all strains. For *msk1Δ* strains, cells were grown under the same conditions used for the OCR assay. For *krs1Δ* strains, cells were grown under shaking in SC medium until OD_600_ = 1.5–2. Cells equivalent to 10 OD_600_ were harvested, and proteins extracted by trichloroacetic acid precipitation, according to a previous protocol (Del Dotto et al., 2018). Proteins corresponding to 1.5 OD_600_ of the initial cells were loaded on 15% sodium dodecyl sulfate‐polyacrylamide gel electrophoresis, and electroblotted onto nitrocellulose filters that were incubated with rabbit anti‐KARS1 polyclonal antibody (ElabScience US, 1:1500 to 1:4000 dilution), mouse anti‐Por1 monoclonal antibody (Abcam, 1:10,000 dilution), and mouse anti‐Pgk1 monoclonal antibody (Abcam, 1:5000 dilution). Blots were incubated with antirabbit secondary antibody (StarBright Blue700, Bio‐Rad, 1:5000 dilution) and antimouse secondary antibody (StarBright Blue520, Bio‐Rad, 1:10,000 dilution), and fluorescent signals were recorded by Chemidoc MP imager (Bio‐Rad). Signals were quantified by Image Lab software (Bio‐Rad), and ratios between mtKARS1 and Por1 or cytKARS1 and Pgk1 were calculated.

For mitochondrial protein synthesis, strains W303‐1B *msk1Δ* transformed with mutant and wild‐type *mtKARS1* alleles were grown in SC medium supplemented with 0.2% glucose and 2% galactose until OD600 = 1.0–1.2 OD. Cells equivalent to 1.2 OD600 were harvested, washed and incubated in 500 µl of 40 mM K‐phosphate buffer pH 6, 2% galactose 2%, with cycloheximide 0.2 µg/ml and 2.5–6 µl of EasyTag™ EXPRESS35S Protein Labeling Mix, [^35^S]‐stabilized aqueous solution (Perkin Elmer) for 10 min. Total extracts were obtained as for western blots, and extract equivalents to 0.5 OD600 were loaded on gel and electroblotted onto nitrocellulose filters. Signals were acquired by autoradiographic Carestream® BioMax® MR film (Kodak).

### 2.3 Statistical analyses

Statistical analyses were performed by using GraphPhPrism8, and Excel. *p* values below .05 were considered statistically significant.

## 3 RESULTS

### 3.1 Clinical features

**Figure 1:**
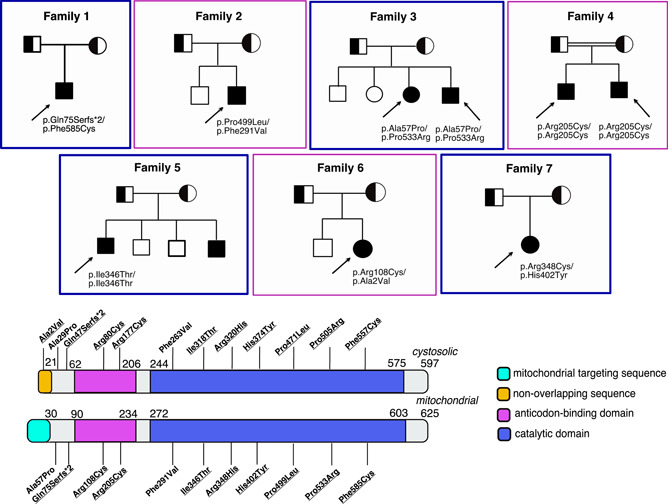
Subjects with biallelic *KARS1* variants and their localization on the two isoforms of *KARS1* protein. Pathogenic variants in bold are novel. The p.(Ala2Val) only affects the cytosolic isoform whereas all remaining variants affect both isoforms. The cytosolic isoform refers to NM_005548.2, NP_001123561.1 (597 amino acids), and the mitochondrial isoform to NM_001130089.1, NP_005539.1 (625 amino acids)

**Figure 2:**
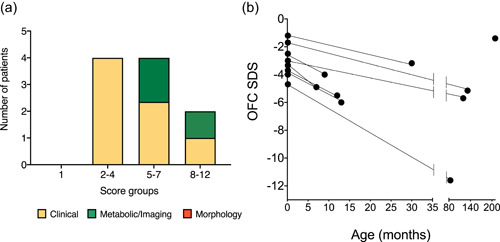
(a) Distribution of mitochondrial disease diagnostic scores among the cases herein reported. Morphology studies were not performed in any of the cases. (b) Standard deviation score (SDS) of the occipitofrontal circumference (OFC) at birth (*n *= 8) and at last evaluation (*n *= 9). OFC at birth was not available for proband 9. OFC SDS at the latest available evaluation is statistically significantly different from birth OFC (*p* < .004)

Nine cases harboring bi‐allelic *KARS1* variants were collected through international collaboration facilitated by Matchmaker exchange (Philippakis et al., 2015), Database of Chromosomal Imbalance and Phenotype in Humans Using Ensembl Resources (DECIPHER) (Firth et al., 2009), GeneMatcher initiative (Sobreira et al., 2015), and the European Reference Network (ERN) ITHACA. ES was performed for all index cases. Their clinical and genetic findings are summarized in Table 1 and Figure 1. Mitochondrial scores were calculated for each case (Table S2) and none had a score less than 2 that is indicative of an unlikely diagnosis of mitochondrial disorder, whereas 22.2% had scores consistent with a possible diagnosis of mitochondrial disorder, 55.5% with a probable diagnosis, and 22.2% with a definitive diagnosis of mitochondrial disorder (Figure 2a). Standard deviation score (SDS) of occipitofrontal circumference (OFC) were recorded at birth and at the latest evaluation for eight and nine individuals, respectively. The average of SDS of OFC at the latest evaluations was significantly reduced compared to OFC at birth (*p* < .004), supporting progressive microcephaly (Figure 2b).

**Table 1:** Demographics and main clinical features of the reported patients’ cohort

	Case 1	Case 2	Case 3	Case 4	Case 5	Case 6	Case 7	Case 8	Case 9	Prevalence in this case series
Gender	Male	Male	Female	Male	Male	Male	Male	Female	Male	7:2 (male:female)
Age average in months (range)	96	16	134	41	25	12	168	10	209	79 (10‐209)
OFC at birth (SDS)	−4.7	−2.3	−3	−1.2	−4	−3.7	−1.7	−3.3	NA	Microcephaly: 6/8 (‐3±1.18) (average±standard deviation)
OFC at last evaluation (SDS)	−11.4	−4	−	−3.2	−6	−5.5	−5.7	−5.2	−1.4	Microcephaly: 8/9 (‐5.2±2.7) (average±standard deviation)
Growth delay	+	+	+	−	+	−	+	+	+	7/9
Developmental delay/Intellectual disability	+	+	+	+	+	+	+	+	+	9/9
Movement disorder	+	+	+	−	+	+	−	−	+	6/9
Seizures	+	+	+	+	+	+	+	−	+	8/9
Oculomotor dysfunction	−	+	+	+	+	+	−	+	NA	6/8
Hearing loss	−	+	−	−	+	−	+	+	+	5/9
Immunological and hematological abnormalities	+	+	+	−	−	−	−	−	NA	3/8
White matter lesions	+	+	−	−	+	+	−	+	+	6/9
Cerebral tissue loss	+	+	−	−	+	+	+	−	+	6/9
Brain calcifications	−	+	−	−	−	−	−	+	+	3/9
Heart involvement	+	−	−	NA	−	−	+	+	−	3/8
Increased lactate (serum, CSF, or MRS)	+	+	NA	NA	+	NA	+	+	+	6/6
*KARS1* pathogenic variant (NM_001130089)(NP_005539.1)	c.223delC, p.Gln75Serfs*2/c.1754T>G, p.Phe585Cys	c.1496C>T, p.Pro499Leu/c.871T>G, p.Phe291Val	c.169G>C, p.Ala57Pro/c.1598C>G, p.Pro533Arg	c.169G>C, p.Ala57Pro/c.1598C>G, p.Pro533Arg	c.613C>T, p.Arg205Cys/c.613C>T, pArg205Cys	c.613C>T, p.Arg205Cys/c.613C>T, pArg205Cys	c.1037T>C, p.Ile346Thr/c.1037T>C, p.Ile364Thr	c.322C>T, p.Arg108Cys/c.5C>T*, p.Ala2Val*	c.1042C>T, p.Arg348Cys/c.1204C>T, p.His402Tyr	

Abbreviations: CSF, cerebrospinal fluid; MRS, magnetic resonance spectroscopy; NA, not available; OFC, occipitofrontal circumference; SDS, standard deviation score.

* The c.5C>T, p.(Ala2Val) only affects the cytosolic isoform NM_005548 and NP_001123561.1, respectively.

#### 3.1.1 Family 1, Case 1

**Figure 3:**
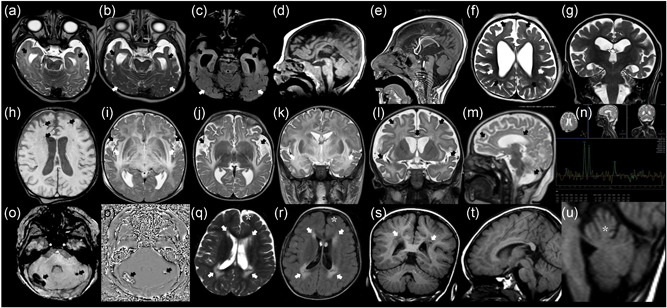
Brain magnetic resonance imaging (MRI) images of case 1 (a)–(g): Axial T2‐weighted images (a), (b), (f), axial FLAIR image (b), sagittal T1‐weighted images (d) and (e), and coronal T2‐weighted image (g) at 5 months (a) and (d) and 4 years (b), (c), (e)–(g). At 5 months the temporal horns and sulci were dilated but the signal of the unmyelinated white matter appeared normal (black arrows in (a)). Note the progressive thinning of the corpus callosum and of a diffuse cerebral tissue loss causing microcephaly between 5 months and 4 years (d) and (e). In the deep temporal, frontal and peritrigonal white matter some subtle hyperintensities appeared between 5 months and 4 years, with a more preserved signal in the posterior white matter (black and white arrows respectively in (b), (c), (f)). Brain MRI images and magnetic resonance spectroscopy (MRS) of case 2 at 12 months (i) and (k) and 17 months (h), (j), (l), and (m), (n): Axial T2‐weighted images (i), (j), axial SWI phase image (h), coronal and sagittal T2‐weighted images (k), (l), and (m). The first MRI scan highlighted bilateral and confluent T2 hyperintensities in basal ganglia, thalamic nuclei, and in capsular, deep, and peripheral white matter causing tissue swelling and sulcal effacement (i) and (k). Note also the prominent involvement of external capsules and of the white matter of temporal lobes (white arrows in (i) and (k)). Incomplete operculization of sylvian fissures was already present in the first MRI and such dilation worsened later (black arrows in (i) and (j)). The severe enlargement of frontal and temporal sulci and the thinning of corpus callosum due to the tissue loss are evident (black arrows in (l) and (m). There is also an arachnoid cyst located inferiorly to the vermis (black arrow in (m)). The reduction of N‐acetyl‐aspartate peak and the presence of a small peak of lactate are shown on MRS (TE 144) (p). Point‐like hypointensities related to calcifications were bilaterally located in the frontal white matter (black arrows in (h)). MRI phase and magnitude SWI images of case 8 (o)–(p)) showed calcifications in the cortex of cerebellar hemispheres (black arrows). Brain MRI images of case 9 (q)–(u)): axial T2‐weighted and FLAIR images (q) and (r), coronal and sagittal T1‐weighted images (s), (t), and (u). Note the bilateral periventricular hyperintensities on T2 and FLAIR images, posteriorly containing little cavities (white arrows in (q), (r), and (s)). Mild enlargement of left frontal subarachnoid spaces and in the superior part of the vermis is also present (asterisks in (q), (r), and (u))

The proband is the only child of healthy nonconsanguineous Caucasian parents with unremarkable family history (Figure 1). He was born by cesarean section after 35 weeks of gestation complicated by intrauterine growth retardation (IUGR). At birth, his weight was 1390 g (<5th centile, −2.7 SDS ‐ adjusted for prematurity), his length 40 cm (<5th centile, −2.6 SDS ‐ adjusted for prematurity) and his head circumference 25.5 cm (<5th centile, −4.7 SDS ‐ adjusted for prematurity). Since the first months of life, he had failure to thrive and from the age of 3 months, he experienced eyelid myoclonus and tonic‐clonic seizures associated with epileptiform abnormalities on EEG. He was started on multiple antiepileptic drugs (pregabalin, clonazepam, and levetiracetam). From 7 months of age, he developed anemia, neutropenia, and hypogammaglobulinemia and was treated with intravenous immunoglobulin. His development was severely delayed: he smiled at 4 months, held his head at 4 years of age, and never achieved the sitting position. On his last evaluation at 4 years and 7 months, his weight was 9 kg (<5th centile, −6.7 SDS), height was 94 cm (<5th centile, −2.8 SDS), and OFC was 35.5 cm (<5th centile, −11.4 SDS) (Figure 2b). He showed sloping forehead, prominent and large ears, thin upper lip, hypotonia, and hypospadia. A brain magnetic resonance imaging (MRI) at 5 months of age showed dilation of supratentorial ventricular system and cerebral subarachnoid spaces, particularly in temporal, sylvian and frontal regions that was associated to mild thinning of corpus callosum and bilateral incomplete hippocampal inversion (Figure 3a,d). Myelination and white matter signal were normal (Figure 3a,d). A control brain MRI at 4 years showed progressive cerebral atrophy, further thinning of the corpus callosum (Figure 3e), and increased enlargement of lateral ventricles and subarachnoid spaces (Figure 3f,g), likely due to progressive and severe cerebral tissue loss, affecting mostly temporal and frontal lobes. In addition, subtle FLAIR and T2 hyperintensities became evident over time in the deep white matter of peritrigonal, temporal, and frontal regions, whereas normal signal of posterior regions was detected (Figures 3b,c and 3f,g). Cerebral magnetic resonance spectroscopy (MRS) showed peak of lactate. Auditory brainstem response, visual evoked potential, and electroretinogram were all normal. His echocardiogram showed a patent foramen ovalis and a mild ventricular septal defect that closed spontaneously. Urinary amino acids showed mild generalized aminoaciduria.

#### 3.1.2 Family 2, Case 2

The proband is the second child of nonconsanguineous Caucasian parents (Figure 1). The mother suffered of cerebral thrombosis at age 21 years. He was born by vaginal delivery after 39 weeks of gestation complicated by IUGR with onset in the III trimester of gestation. At birth, his weight was 2550 g (1st centile, −2.3 SDS), his length 50 cm (35th centile, −0.4 SDS), and his head circumference 32 cm (1st centile, −2.3 SDS). His hearing screening revealed abnormal hearing and he was later diagnosed with bilateral sensorineural hearing loss. Echocardiogram was normal. At 12 months, he developed respiratory failure during acute gastroenteritis and a few days after the MMR immunization. A brain MRI showed supratentorial bilateral and confluent FLAIR and T2 hyperintensities in deep and peripheral white matter, especially involving external capsules, temporal and frontal lobes, and causing tissue swelling and diffuse brain sulcal effacement. Moreover, bilateral involvement of basal ganglia and thalamic nuclei was observed (Figure 3i,k). Incomplete operculization of sylvian fissures and a posterior fossa arachnoid cyst were also detected (Figure 3i,m). He developed anemia requiring red blood cell transfusion and severe hypogammaglobulinemia, hypoalbuminemia, and coagulopathy. He had two episodes of pneumonia at 15 and 17 months of life. He developed tonic‐clonic seizures at 16 months of life and phenytoin, phenobarbital, clobazam, and topiramate were used to control seizures. He was also treated with baclofen for spasticity. He had pendular and saccadic nystagmus with difficulties in right lateral deviation. A control brain MRI performed at 17 months of age showed a reduction of supratentorial white matter hyperintensities but thinning of corpus callosum and severe dilation of ventricles and subarachnoid spaces were detected (Figure 3j,l). Severe reduction of *N*‐acetyl‐aspartate was evident in the affected white matter on Single Voxel MRS in association to a small peak of lactate (Figure 3n). Some point‐like hypointensities on Susceptibility Weighted Imaging (SWI) suggesting calcifications were evident in bilateral frontal white matter (Figure 3h). At 9 months of age, he had not yet achieved the sitting position and his growth parameters were all below the 3rd centile.

#### 3.1.3 Family 3, Cases 3 and 4

Proband 3 is the third child of healthy nonconsanguineous Caucasian parents (Figure 1). She was born after 40 weeks of gestation with a birth weight of 2700 g (<5th centile, −1.7 SDS), length 47 cm (<5th centile, −1.6 SDS), and head circumference 31 cm (<5th centile, −3 SDS). Vertical gaze palsy was noted in the first months of life. She sat independently at about 16 months of age, walked independently at 3 years but she was non‐verbal up to the age of 11 years, although she followed simple commands. Her first seizures occurred at 10.5 years and she was started on levetiracetam with no recurrence of the seizures. Echocardiogram was normal. A brain MRI performed in the first year of life failed to detect any abnormality. At her last evaluation at 11 years of age, her weight was 30.6 kg (12th centile, −1 SDS), height was 133.3 cm (5th centile, −1.6 SDS), and OFC was 47.5 cm (<5th centile, −5 SDS) (Figure 2b). Both vertical and horizontal nystagmus were evident, and she had mild dysmorphic features including a triangular face, thin upper lip, short philtrum, broad nose, tapering, and long fingers. This girl has been previously reported in a series of re‐evaluated cases (case#10 in the previous publication by Cope et al., 2020)). She had a younger brother (case 4) who was born via vaginal delivery after 39 weeks of uncomplicated gestation. At birth, his weight was 3550 g (50th centile, 0 SDS), length 50.8 cm (47th centile, −0.1 SDS), and OFC 33.5 cm (11th centile, −1.2 SDS). Vertical nystagmus was noted in the first few months of life and at 2.5 years, roving eye movements without tracking, unconjugated gaze, and nystagmus were also observed. He had swallowing difficulties and was also noted to have increased muscle tone, especially at the ankles. At 11 months, he developed infantile spams that were treated with ACTH, and later with vigabatrin and zonisamide. At the age of 3 years, he could roll over, sit independently for short periods, and could say two words. At his latest evaluation at 30 months of age, his OFC was 44.5 cm (<5th centile, −3.2 SDS) and at the age of 3.4 years his weight was 13.7 kg (20th centile) and his height 95.9 cm (19th centile).

#### 3.1.4 Family 4, Cases 5 and 6

Proband 5 was the second child of healthy consanguineous (first‐cousins) parents from the Middle East with unremarkable family history (Figure 1). Prenatal and perinatal history were uncomplicated. His birth weight was 2385 g (<5th centile, −2.6 SDS) and OFC was 30 cm (<5th centile, −4 SDS). Developmental milestones were delayed and at 2 years he offered eye contact, but without tracking and he had no intentional movements. His muscle tone was increased with brisk reflexes. He was found to have hearing loss. He had feeding difficulties and failure to thrive. At 25 months, his weight was 9.1 kg (<5th centile, −2.7 SDS), height 85.5 cm (19th centile), and OFC 39 cm (<5th centile, −6 SDS) (Figure 2b). At the age of 9 months, he developed infantile spams that were responsive to vigabatrin. Echocardiogram was normal. A brain MRI at 3 months revealed no abnormalities and showed mild cerebral atrophy with thin corpus callosum and lack of myelination at 2 years. On cerebral MRS lactate was slightly elevated. Proband 6 is the younger brother of proband 5 and his prenatal and perinatal history were also uncomplicated. His birth weight was 2930 g (8th centile) and his OFC was 30.5 cm (<5th centile, −3.7 SDS). He was more alert than his brother, offered eye contact and had eye tracking but as his older brother, he had random eye movements and nystagmus. He achieved no other developmental milestones. He had increased muscle tone with brisk reflexes. At the age of 9 months, he developed infantile spasms that did not respond to vigabatrin, phenobarbital, levetiracetam, and topiramate. He had feeding difficulties and failure to thrive. At the age of 12 months, his height was 76 cm (54th centile), weight 8.5 kg (12nd centile), and OFC 39 cm (<5th centile, −5.5 SDS). A bran MRI at 7 months showed cerebral atrophy with thin corpus callosum and lack of myelination. Cerebral MRS was normal. On physical exam, he was not noted to have murmurs and he has not been evaluated by echocardiogram.

#### 3.1.5 Family 5, Case 7

Proband 7 is a 14‐year‐old male born to healthy non‐consanguineous Hispanic parents (Figure 1). Family history was remarkable for a 3‐year‐old brother born with ambiguous genitalia and normal development and a 2‐year‐old brother with ambiguous genitalia and severe developmental delay. He was born at term of normal gestation with a birth weight of 2765 g (10th centile, −1.3 SDS), length 48 cm (21st centile, −0.8 SDS) and OFC 32 cm (4th centile, −1.7 SDS). He was also noted to have ambiguous genitalia that was surgically corrected. Auditory brainstem response at 12 months was consistent with bilateral profound sensorineural hearing loss. At 15 months of age, he developed seizures that were treated with topiramate and ketogenic diet with partial response. He developed failure to thrive, hypotonia, spastic quadriplegia, and contractures by the age of 2 years. At 11 months of age, a brain MRI scan showed enlargement of cerebrospinal fluid spaces and ventricular system enlargement and thinning of corpus callosum. The echocardiogram revealed a patent foramen ovale. At the age of 10 years, he was nonambulatory and nonverbal. At the same age, his weight was 21.8 kg (1st centile, −2.4 SDS), height was 121 cm (<5th centile, −2.7 SDS), and OFC was 45.5 cm (<5th centile, −5.7 SDS) (Figure 2b). Trio‐ES revealed a known pathogenic hemizygous splicing variant in the *AR* gene (NM_000044.4): c.2667C>T (p.Ser889=) (Hellwinkel et al., 2001), consistent with the diagnosis of partial androgen insensitivity syndrome. The *AR* variant was also detected in his younger sibling with ambiguous genitalia and normal development whereas the youngest sibling with ambiguous genitalia and developmental delay was not tested.

#### 3.1.6 Family 6, Case 8

The proband is the second child of healthy nonconsanguineous Caucasian parents with unremarkable family history (Figure 1). She was born after 37 weeks of gestation complicated by IUGR. At birth, her weight was 2010 g (<5th centile, −3.6 SDS), length 41 cm (<5th centile, −4.7 SDS), and OFC 31 cm (<5th centile, −3.3 SDS). By 5–6 weeks of life, she was noted to have hypotonia, feeding difficulties, and failure to thrive. Intermittent strabismus, visual upward fixation, random eye movements, and lack of eye contact were noted (Supplementary Video). She achieved head control at 5 months of age. At 10 months, her length was 67.0 cm (<5th centile, −2.2 SDS), weight 6.3 kg (<5th centile, −3.0 SDS) and OFC 39 cm (<5th centile, −5.2 SDS) (Figure 2b). Echocardiogram revealed an atrium septal defect. Brain SWI at 9 months identified two hypointense areas in the cortex of the cerebellar hemispheres whose signal inverted on magnitude map, and therefore were related to calcifications (Figure 3 o,p). Increased lactate was detected on cerebral MRS and blood. Initial auditory brainstem response was normal but re‐testing at 10 months showed bilateral sensorineural hearing loss with thresholds of 25–45 dB.

#### 3.1.7 Family 7, Case 9

The proband is the only child of healthy nonconsanguineous Caucasian parents with unremarkable family history (Figure 1). He was born after 40 weeks of uncomplicated gestation. At birth, his weight was 3855 g (85th centile, +2 SDS), her length 60 cm (>97th centile, >+3 SDS). Birth OFC measurement was unavailable. At 6 months of life, he was noted to have hearing loss, hypotonia, and developmental delay. At age 6 years, he gradually lost the acquired gross motor skills, and became unable to crawl, walk, and speak. Moreover, he developed tonic‐clonic seizures and was started on oxcarbamazepine and clonazepam with good control of the seizures. At the last clinical evaluation at 17.4 years, his weight was 38.6 kg (<5th centile, −4.3 SDS), height 149 cm (<5th centile, −3.5 SDS), and OFC 54.4 cm (7th, −1.4 SDS) (Figure 2b). A brain MRI performed at 24 months of age showed T2 hyperintensities in the posterior thalami and in periventricular (especially peritrigonal) white matter where small cavitations were also detected (Figure 3q–s). Mild enlargement of subarachnoid spaces of left frontal region and superior portion of the vermis was also noted (Figures 3q,r and 3t,u). A head CT scan showed with bilateral periventricular basal ganglia calcifications. The echocardiogram was normal.

### 3.2 KARS1 variants

**Figure 4:**
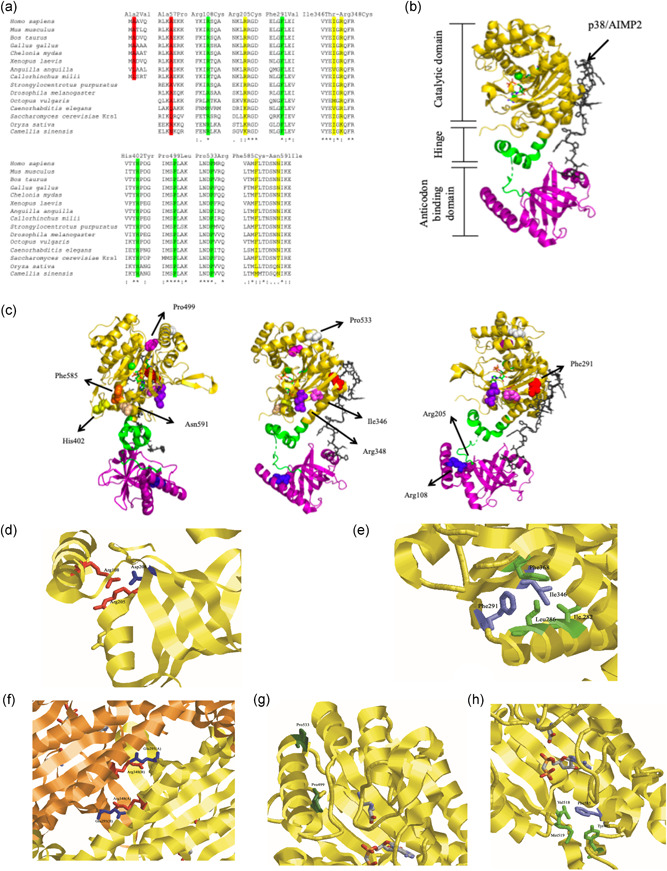
(a) Aligned regions around mutated amino acids. Human mitochondrial and cytosolic LysRS were aligned with LysRS from other organisms, including mammals, birds, reptiles, amphibians, bony fishes, cartilaginous fishes, echinoderms, arthropods, mollusks, fungi, monocotyledons, and dicotyledons. Conserved amino acids are highlighted in green, semi‐conserved amino acids are highlighted in yellow, amino acids conserved only in some organisms are highlighted in red. (b) Crystal structure of of LysRS (PDB: 4dpg) was loaded into PyMOL. The anticodon binding domain is indicated in purple while the catalytic domain in yellow. (c) The amino acid residues affected by variants found in cases are shown on the three different images of KARS1‐p38 complex. (d)–(h) Crystal structure of LysRS around amino acids which are mutated. Amino acids are represented as sticks and the protein is reported as cartoon. (d) Region around Arg108 and Arg205, in red, with Asp208 in blue. (e) Region around Phe291 and Ile346, in magenta, with Ile182, Leu286, and Phe368 in green. (f) Arg348, in red, in both LysRS subunits, with Glu295 in blue. (g) Pro499 and Pro533, in the dark green, in the catalytic domain with Lys and AMP as sticks in CPK colors. (h) Phe585, in magenta, with Tyr401, Val518, and Met519 in green

Twelve *KARS1* variants were identified in the nine cases herein reported: p.(Ala2Val) (compound heterozygous), p.(Ala57Pro) (compound heterozygous), p.(Gln75Serfs*2) (compound heterozygous), p.(Arg108Cys) (compound heterozygous), p.(Arg205Cys) (homozygous), p.(Phe291Val) (compound heterozygous), p.(Ile346Thr) (homozygous), p.(Arg348Cys) (compound heterozygous), p.(His402Tyr) (compound heterozygous), p.(Pro499Leu) (compound heterozygous), p.(Pro533Arg) (compound heterozygous), and p.(Phe585Cys) (compound heterozygous) (Figure 1). Nine out the 12 variants (p.(Ala2Val), p.(Gln75Serfs*2), p.(Arg108Cys), p.(Arg205Cys), p.(Arg348Cys), p.(His402Tyr), p.(Pro499Leu), p.(Pro533Arg), and p.(Phe585Cys)) have not been previously reported. All variants are very rare in controls (MAF<0.001 in GnomAD) and alignment with LysRS showed that mutated most amino acids are either highly conserved (Arg108, Phe291, His401, Pro499, and Pro533) or semi‐conserved (Arg205, Ile346, Arg438, Phe585, and Asn591) in mammals, fungi, and plants. The Ala57 residue is conserved in most animals, whereas the Ala2 is conserved in most tetrapods but not in fishes and invertebrates, in which the program failed to align the N‐terminal region of cytosolic LysRS (Figure 4a).

Pathogenicity scores predicted *KARS1* variants to be predominantly damaging (Table S3) and all of them except for p.(Ala2Val) were classified as pathogenic or likely pathogenic according to ACMG criteria (Richards et al., 2015) (Table S3). LysRS protein consists of the anticodon binding domain and catalytic domain, link by a hinge (Figures 1 and 4b). Most amino acids affected by variants localized to the catalytic domain (Phe291, Ile346, Arg348, His402, Pro499, Pro533, Phe585, and Asn591) or the anticodon binding domain (Arg108 and Arg205) (Figure 4c). Arg108 located in the α‐helix at the N‐terminal part closed to Arg205, forms a salt bridge with Asp208 (Figure 4d). Arg205 could form a hydrogen bond with the carbonyl group of the backbone (Figure 4d). Both Arg108 and Arg205 residues appear to be important in the maintenance of local conformation of the anticodon binding domain. Phe291 localizes at the LysRS‐p38 interaction interface and a previous study reported that substitution with a valine residue reduces this interaction, thus impairing the association of LysRS with the MSC (Scheidecker et al., 2019). The Ile346 is close to Phe291 and together with other amino acids (Ile282, Leu286, and Phe368) form a hydrophobic pocket near to the dimerization interface (Figure 4e). Therefore, variants affecting these amino acids could affect dimerization. The same consideration can be made for Arg348, which forms a salt bridge with Glu295 amino acid presents in the reciprocal LysRS (Figure 4f). Pro499 and Pro533 are localized at the beginning of two α‐helix within the catalytic domain but distant from the active site, and thus variants on these residues may affect the secondary structure of that domain (Figure 4g). Phe585 is part of a hydrophobic pocket along with Tyr401, Val518, and Met519 residues that are all relatively close to the active site, and thus variants at this residue could compromise the structure and secondarily, the aminoacylation reaction (Figure 4h).

### 3.3 Functional studies in yeast

Functional consequences of all *KARS1* missense variants (p.(Ala2Val), p.(Ala57Pro), p.(Arg108Cys), p.(Arg205Cys), p.(Phe291Val), p.(Ile346Thr), p.(Arg348Cys), p.(His402Tyr), p.(Pro499Leu), p.(Pro533Arg), and p.(Phe585Cys)) plus the p.(Asn591Ile) variant recently found in a patient with microcephaly (Boonsawat et al., 2019), were investigated in *Saccharomyces cerevisiae*. While human *KARS1* encodes both the cytosolic and mitochondrial isoforms of LysRS, in yeast two different genes, namely *KRS1* and *MSK1* encode for the cytosolic LysRS (cytLysRS) and mitochondrial LysRS (mtLysRS), respectively. Therefore, we investigated the effects of both *mtKARS1* and *cytKARS1* mutants separately, through heterologous complementation of human *mtKARS1* and *cytKARS1* in strains deleted for *MSK1* and *KRS1*, respectively.

**Figure 5:**
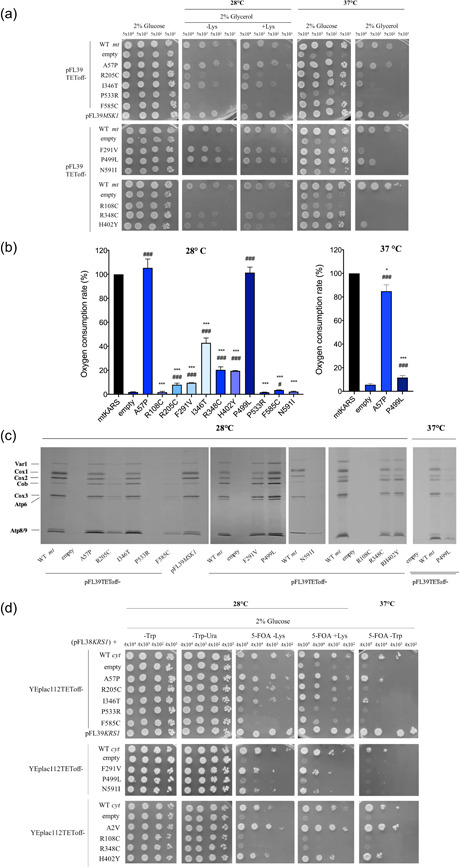
(a) Oxidative growth phenotype of haploid *msk1*Δ strains transformed with *mtKARS1* wild‐ type or mutant alleles cloned in pFL39‐TEToff, or *MSK1* cloned in pFL39. Growth assays (10‐fold dilution spots starting from 5 × 10^4^ cells/spot) were performed in SC medium supplemented with the indicated carbon sources at 28°C and 37° C, and pictures were taken after 2–5 days of growth. The one‐letter nomenclature used in yeast has been utilized. (b) Respiratory activity of the same strains reported in (a). Oxygen consumption rate (OCR) was measured on at least four independent clones. OCR was normalized to the strain transformed with the *mtKARS1* wild‐type allele. Values are means ± standard deviation. *p < .05; *** *p* < .001 relative to *mtKARS1* wild‐type strain and ^#^ *p* < .05; ^###^ *p* < .001 relative to empty plasmid strain using ANOVA followed by a Bonferroni's post hoc test. (c) Representative images of the mitochondrial protein synthesis of the strains reported in (a) at 28°C and 37°C. Experiments were performed on three independent clones for each mutant. (d) Viability of haploid *krs1Δ* strains transformed with *cytKARS​1* wild‐type or mutant alleles cloned in YEplac112‐TEToff, or *KRS1* cloned in pFL39. Growth assays were performed growing cells in liquid SC medium supplemented with uracil for 24 h until the early stationary phase and plating them (10‐fold dilution spots starting from 4 × 10^5^ or 4 × 10^4^ cells/spot) in SC medium supplemented with 2% glucose, with or without 5‐FOA, at the indicated temperatures, and pictures were taken after 2–4 days of growth

Previous studies have shown that human cDNA encoding *KARS1* isoform 1 (*mtKARS1*, accession number NM_001130089.1) complements *MSK1* deletion in yeast (Sepuri et al., 2012). Consistent with previous findings, a transformation of wild‐type human *mtKARS1* cloned in a single copy plasmid under the TET‐off promoter restored growth defect of the *msk1Δ* strain. The *mtKARS1* mutant alleles were introduced into *msk1Δ*, carrying a wild‐type *MSK1* on a *URA3*‐bearing vector, which is lost upon exposure to 5‐FOA. Oxidative growth was then determined through spot assay on glycerol medium and most *mtKARS1* variants had negative effects on growth (Figure 5a) with the p.(Arg205Cys), p.(Phe585Cys), p.(Arg108Cys), p.(Pro533Arg), and p.(Asn591Ile) showing the more severe defect. The latter three mutants were completely unable to restore oxidative growth and behaved as *null* alleles. When growth was scored at 37°C, also Ala57Pro and Pro499Leu mutants showed a mild reduction in growth (Figure 5a). The growth defects were similar in medium without lysine or supplemented with 50 µg/ml lysine, suggesting that lysine is not a limiting factor. Consistently, OCR was affected for most mutants, and the p.(Arg108Cys), p.(Pro533Arg), and p.(Asn591Ile) mutants were similar to the strain transformed with the empty plasmid, whereas p.(Arg205Cys), p.(Phe291Val), p.(Arg348Cys), p.(His402Tyr), and p.(Phe585Cys) mutants showed activities ranging between 10% and 20% of wild‐type, and the p.Ile346Thr of about 40% (Figure 5b). The p.(Ala57Pro) and p.(Pro499Leu) mutants resulted in decreased OCR at 37°C by 25% and 85% respectively, according to the oxidative growth phenotype. Consistent with the OCR defect, strains transformed with *mtKARS1* harboring the p.(Arg205Cys), p.(Phe291Val), p.(Arg348Cys), p.(His402Tyr), p.(Phe585Cys) and p.(Asn591Ile) variants showed markedly reduced synthesis of mitochondrial protein (Figure 5c). Levels of mitochondrial proteins were severely affected by the p.(Arg108Cys) and p.(Pro533Arg) variants whereas strains expressing the p.(Ala57Pro) and p.(Ile346Thr) showed mild decrease, particularly for cytochrome c oxidase (Cox) subunits 1 and 2. Yeast transformed with the p.(Pro499Leu) showed no changes at 28°C but decreased mitochondrial protein levels at 37°C. To evaluate whether the oxidative defects due to *mtKARS1* mutants are caused by protein instability, steady‐state protein levels of mutant LysRS were measured. Protein levels of mutant mtLysRS normalized to Por1 levels were similar to wild‐type mtLysRS (between 70% and 130%), except for p.(Arg108Cys) and p.(Arg348Cys) mutants that showed 40‐50% decrease compared with wild‐type (Figure S1A). These results indicated that the partial or total mitochondrial function impairment was mainly caused by deficiency of enzyme activity rather than reduced protein levels.

To investigate the functional consequences of *KARS1* variants on the activity of cytLysRS, cDNA of *KARS1* isoform 2 (*cytKARS1*; NM_005548.2) was first cloned into the same centromeric expression vector and inserted in a *krs1*Δ strain expressing the wild‐type *KRS1* on a *URA3*‐bearing vector. This construct was unable to rescue the lethal phenotype of *krs1Δ* since no growth was observed in medium supplemented with 5‐FOA, on which only cells which have lost yeast *KRS1* could grow (data not shown). However, when subcloned into the multicopy vector, YEplac112TET‐off, *cytKARS1* ORF was able to support the growth on medium supplemented with 5‐FOA, though at a lesser extent that yeast *KRS1* under its endogenous promoter. The *cytKARS1* cDNA was then mutagenized to introduce the missense variants found in patients and inserted into the *krs1Δ* strain. Constructs expressing the missense variants Arg108Cys, Arg348Cys, Pro499Leu, and Pro533Arg were unable to rescue the growth defect of *krs1Δ* mutants (Figure 5d). Strains expressing the Arg205Cys, Phe291Val, Ile346Thr, Phe585Cys, and Asn591Ile variants showed reduced growth of variable severity, whereas those expressing Ala57Pro and His402Tyr variants showed a mild growth defect only at 37°C (Figure 5d). The p.(Ala2Val) variant affects a residue only present on cytLysRS (Figure 5d). Unexpectedly, *krs1Δ* strain expressing p.(Ala2Val) mutant exhibited increased growth compared with parental *cytKARS1* at both 28°C and 37°C. To investigate whether the p.(Ala2Val) variant indirectly impacts mitochondrial function in *krs1Δ* strain expressing this variant, oxidative growth and OCR were investigated, but they were found to be both similar to wild‐type (data not shown).

For most mutants, the growth defects were not improved by supplementation of lysine in medium (Figure 5d). However, the p.(Phe585Cys) and to a lesser extent the p.(Pro533Arg) showed partial growth improvement upon lysine supplementation, suggesting that cytoplasmic lysine levels are a limiting factor for the activity of these two *cytKARS1* mutant alleles.

Steady‐state levels of mutant LysRS normalized for Pgk1 were all similar to wild‐type cytLysRS (between 80% and 120%) except for the p.(Ala2Val) that showed a 2‐fold increase (Figure S1b). As for their mitochondrial counterparts, these results indicated that the growth impairment of cytLysRS mutants was mainly caused by deficiency of enzyme activity rather than protein levels. We speculate that the improved growth of p.(Ala2Val) mutant can be due to the higher steady‐state protein levels.

Although p.(Arg205Cys), p.(His402Tyr), and p.(Asn591Ile) variants strongly or totally abolished growth of *mtKARS1* mutant strains on oxidative carbon sources, they induced only a moderate growth defect on *cytKARS1* mutant strains, suggesting that these variants have a greater effect on the mitochondrial isoform compared to the cytosolic isoform. In contrast, p.(Pro499Leu) and p.(Arg348Cys) variants did not affect the growth of *mtKARS1* mutant strain at 28°C but they severely affected the growth of *cytKARS1* mutant strain at the same temperature, suggesting these variants have greater effects on the cytosolic isoform compared with the mitochondrial isoform.

## 4 DISCUSSION

We report on clinical and neuroradiologic findings of nine individuals carrying bi‐allelic *KARS1* variants and the functional validation of the identified missense variants in yeasts. Progressive neurological signs were observed in most of the patients and included developmental delay/intellectual disability (9/9), microcephaly (8/9), seizures (7/9), and oculomotor dysfunction (6/8). Brain abnormalities involving gray and/or white matter have been recognized in individuals with *KARS1*‐related disorder (Itoh et al., 2019). In our series, neuroimaging studies showed that *KARS1* pathogenic variants can result in a wide range of anomalies including white matter signal changes and severe cerebral atrophy with thinning of corpus callosum. White matter signal anomalies were observed in 6/9 subjects and more prominent white matter lesions were observed in two of these six cases, consistent with previous reports (Ardissone et al., 2018; Itoh et al., 2019; van der Knaap et al., 2019),while signs of progressive cerebral loss were more prevalent in our case‐series (6/9) compared with previously described cases. LysRS are involved in myelin formation and defects in other ARSes (e.g., *AARS2*, *DARS2*, *EARS2*, and *LARS2*) have been found to be responsible for leukodystrophy (van der Knaap et al., 2019). Consistent with the previous report of white matter signal anomalies with basal ganglia calcifications (Ardissone et al., 2018), our cases 2 and 8 showed bilateral calcifications in frontal deep white matter and cerebellar hemispheric cortex, respectively. In case 9, basal ganglia calcifications were detected by CT scan in the periventricular region. Interestingly, pathogenic variants in mit‐tRNA^Lys^ (*MTTK*) gene encoding the mitochondrial tRNA for lysine have also been associated with leukodystrophy with basal ganglia calcifications (Kisler et al., 2010), suggesting that mitochondrial cytopathy might be the underlying defect for these neuroradiological abnormalities. Severe microcephaly has already been described in individuals with bi‐allelic *KARS1* pathogenic variants (McMillan et al., 2015; Murray et al., 2017), but we found that the microcephaly and signs of cerebral tissue loss (including thinning of corpus callosum) are progressive, suggesting neurodegeneration.

Pendular nystagmus, ophthalmoplegia, jerky eye movements are typical clinical signs of mitochondrial diseases (Schrier & Falk, 2011) and have been reported in individuals with *KARS1* defects (Ardissone et al., 2018; Fuchs et al., 2019; Itoh et al., 2019; Joshi et al., 2016; Kohda et al., 2016; Lieber et al., 2013; McMillan et al., 2015). They appear to be relatively frequent also in our series (6/8) and might be a diagnostic clue.

LysRS affects the expression of several genes involved in the immune system (Carmi‐Levy et al., 2011; Kwon et al., 2019; Park et al., 2005; Yannay‐Cohen et al., 2009). Consistent with these function, immuno‐hematologic disorders, including microcytic anemia, thrombocytopenia, neutropenia, pancytopenia, defects of T lymphocytes, and hypogammaglobulinemia have been reported in *KARS1* disease (Ardissone et al., 2018; Fuchs et al., 2019; Itoh et al., 2019; Kuki et al., 2011; Murray et al., 2017; Yoshimura et al., 1997). Hypogammaglobulinemia and anemia were found in cases 1 and 2 of our series. Moreover, postvaccination acute encephalomyelitis was the presenting feature of case 2 in this series. In previous reports, patients with *KARS1*‐related disorder showed neurological symptoms that worsened after mild infections (Itoh et al., 2019).

LysRS is a combined cytosolic and a mitochondrial ARS, but as GlyRS defects (McMillan et al., 2014; Nafisinia et al., 2017), its defect is not included among the causes of mitochondrial diseases. However, by mitochondrial disease diagnostic scores based on clinical, biochemical, and morphology criteria (Morava et al., 2006), a diagnosis of mitochondrial disease was established for all cases of this cohort, notwithstanding those biochemical investigations were available for 7/9 cases and morphology studies in none of them.

The functional studies in yeasts of the missense variants on both the mitochondrial and the cytoplasmic isoforms allowed to dissect the consequence of each variant on mitochondrial and cytosolic functions of the LysRS. A yeast growth defect was detected for most variants and none of the variants uniquely affected one isoform, with the exception of the p.(Ala2Val) that is only present in the cytosolic isoform. However, the cytosolic p.(Ala2Val) variant did not display decreased function, but rather seemed to increase the growth rate of the *krs1Δ* strain. Therefore, the pathogenic role of this variant remains uncertain. For several variants, a variable degree of impairment was observed when the same variant was expressed by cytLysRS or mtLysRS, suggesting that defects of both cytLysRS or mtLysRS contribute to the phenotype. Nevertheless, the contribution of each defective isoform to the spectrum of clinical manifestations is difficult to untangle because they are largely present in the compound heterozygous state (6/9).

Patients carrying pathogenic *KARS1* variants have been treated with mitochondrial cocktail or idebenone (Avula et al., 2014; Verrigni et al., 2017) that is expected to have some effects only on manifestations dependent on mitochondrial dysfunction rather than on the defect of the cytosolic isoform. For therapy of patients with various *ARS* defects, supplementation of the corresponding amino acid or high protein intake has been proposed (Casey et al., 2015; Fuchs et al., 2019) based on in vitro studies showing a variable degree of biochemical improvements (Friederich et al., 2018; Hadchouel et al., 2015). Similarly, supplementation with lysine might provide benefit in patients carrying *KARS1* pathogenic variants. In the present study, the detrimental effects of two *cytKARS1* variants located in the catalytic domain, namely p.(Phe585Cys) and p.(Pro533Arg) expressed in yeast, were partially improved by lysine supplementation. Interestingly, the Pro533 is near the Glu529 corresponding to the Glu428 in *E. coli* LysRS that is directly involved in the binding to lysine (Onesti et al., 1995). Moreover, the Phe585 is in close proximity to the Arg581 corresponding to the Arg480 in *E. coli* LysRS that binds through an ionic interaction the gamma‐phosphate of ATP during the synthesis of the intermediate lysyl‐adenylate (Desogus et al., 2000). Therefore, variants affecting Pro533 and Phe585 could alter the local structure of the lysine binding domain, resulting in decreased binding affinity for lysine, thus explaining the growth improvement after lysine supplementation. It remains to be determined whether lysine supplementation would be effective also in higher eukaryotes. Nevertheless, lysine supplementation that is currently administered to patients with lysinuric protein intolerance is sufficiently safe to be investigated in clinical trials.

In conclusion, *KARS1*‐related disorder is a multi‐system mitochondrial disease with congenital progressive microcephaly and cerebral tissue loss, white matter anomalies, epilepsy, oculomotor dysfunction, and immune‐hematological dysfunctions. Therefore, we expand the spectrum of clinical abnormalities associated with *KARS1* pathogenic variants and emphasize the importance of mitochondrial ​and cytosolic LysRS dysfunction in the pathogenesis of this disorder.

## CONFLICT OF INTERESTS

All the authors declare that there are no conflicts of interests.

## Supporting information

Supporting information.

**Supplementary video**. Ocular movement disorder characterized by upward fixation, random eye movements in individual 8.

## Data Availability

*KARS1* pathogenic variants data were deposited to LOVD database (https://databases.lovd.nl/shared/variants/KARS/unique).
