# Supplementary material for: Bi‐allelic KARS1 pathogenic variants affecting functions of cytosolic and mitochondrial isoforms are associated with a progressive and multisystem disease
Source: Hum Mutat. 2021 May 11;42(6):745–61. doi: 10.1002/humu.24210 (PMC8251883; doi:10.1002/humu.24210)
Supplement: Supplementary file 1 — Supporting information. [file HUMU-42-745-s001.docx]

**Supplementary material**

**Bi-allelic *KARS1* pathogenic variants affecting functions of cytosolic and mitochondrial isoforms are associated with a progressive and multi-system disease.**

**Authors:** Gerarda Cappuccio, Camilla Ceccatelli Berti, Enrico Baruffini, Jennifer Sullivan, Vandana Shashi, Tamison Jewett, Tara Stamper, Silvia Maitz, Francesco Canonico, Anya Revah-Politi, Gabriel S. Kupchik, Kwame Anyane-Yeboa, Vimla Aggarwal, Andreas Benneche, Eirik Bratland, Siren Berland, Felice D’Arco, Cesar Augusto Alves, Adeline Vanderver, Daniela Longo, Enrico Bertini, Annalaura Torella, Vincenzo Nigro, Telethon Undiagnosed Diseases Program, Alessandra D’Amico, Marjo S. van der Knaap, Paola Goffrini, and Nicola Brunetti-Pierri

**
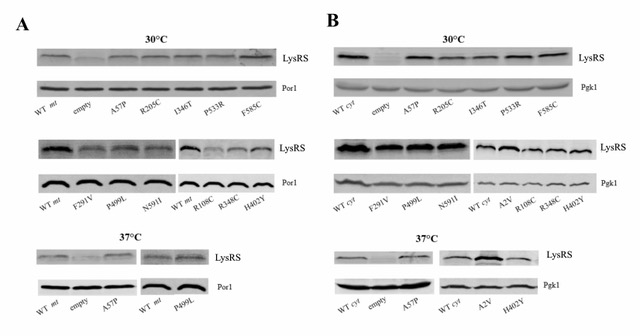
Supplementary Figure 1.** **(A-B)** Western blot assays on *mtKARS*- and *cytKARS*-transformed strains as reported in Material and Methods. **(A)** Representative image of a Western blot of proteins extracted from haploid *msk1Δ* strains transformed with pFL38*MSK1* and with *mtKARS* wild type or mutant alleles. A slightly lower non-specific band was observed in strain transformed with the empty plasmid, likely due to endogenous Krs1 protein, which accounts for less than 5% of the total signal. **(B)**Representative image of a Western blot of proteins extracted from haploid *krs1Δ* strains transformed with pFL38*KRS1* and with *cytKARS* wild type or mutant alleles, and quantification relative to Pgk1 protein normalized to the transformed with the *cytKARS* wild type allele. A non-specific band at the same levels of *cytKARS* was observed in strain transformed with the empty plasmid, likely due to endogenous Krs1 protein, which accounts for less than 3% of the total signal. Proteins extraction was performed at temperatures indicated in the pictures.

**Supplementary Table 1.** Primers for yeast studies.

| **Oligo** | **Sequence** | **Use** |
| --- | --- | --- |
| MSK1CKpnFw | GGGGGGGTACCGACTTGTACACGCACATACC | Cloning |
| MSK1CHindRv | GCGGCAAGCTTCCATTTAATCGTATCAATTTGGTG | Cloning |
| MSK1S1Fw | GTGGGAGTAGTGAGGATAAG | Sequencing |
| MKS1DFw | CTCTTCGCCGAGATAAATATAC | Gene disruption |
| MSK1DRv | GTTTTTCGACACTCTCATTAGC | Gene disruption |
| KRS1CKpnFw | GCCCCGGTACCCACATTTTAGCGTATTTCTGAGC | Cloning |
| KRS1CPstRv | GCCCCCTGCAGAAACACGATCCATTTGAAGC | Cloning |
| KRS1S1Fw | GAAATGACTCTTAATGAACTTCG | Sequencing |
| KRS1S2Fw | CAGAGTGCAATTATTGACACC | Sequencing |
| KRS1DFw | CTCTGTCCTTCCTCCGAGAAATCCGTAATAAACTTCAATAGCATAGCTTCGTACGCTGCAGGTCGACG | Gene disruption |
| KRS1DRv | AATAATAATACTTACATTATACAATATGTTCTAATATCTTTTAGCGATATCATCGATGAATTCGAGC | Gene disruption |
| KARSiso1CFw | CATGATGCGGCCGCACACAATGTTGACGCAAGCTGCTG | Cloning |
| KARSiso2CFw | CATGATGCGGCCGCACACAATGGCAGCAGTGCAGGCGGC | Cloning |
| KARSCRv | CATGATGCGGCCGCTTAGACAGAAGTGCCAACTGTTGTG | Cloning |
| KARSS1Fw | GTGAGCTGAGCATCATTCC | Sequencing |
| KARS2A2VFw | GCCGCACACAATGGTAGCAGTGCAGGCGGCCGAGG | Mutagensis |
| KARS2AV2Rv | CCTCGGCCGCCTGCACTGCTACCATTGTGTGCGGC | Mutagensis |
| KARSA57PFw | GCTGAAGAGACGCCTGAAACCGGAGAAGAAAGTAGCAGAGAAGG | Mutagensis |
| KARSA57PRv | CCTTCTCTGCTACTTTCTTCTCCGGTTTCAGGCGTCTCTTCAGC | Mutagensis |
| KARSR108CFw | CCAAATCAATACTACAAAATCTGCAGTCAAGCAATTCATCAGC | Mutagensis |
| KARSR108CRV | GCTGATGAATTGCTTGACTGCAGATTTTGTAGTATTGATTTGG | Mutagensis |
| KARSR205CFw | GAATTTATTCATATTAATAACAAACTCTGCAGGGGAGACATAATTGG | Mutagensis |
| KARSR205CRv | CCAATTATGTCTCCCCTGCAGAGTTTGTTATTAATATGAATAAATTC | Mutagensis |
| KARSF291VFw | GAAGTTTCTTAGATGAGCTGGGAGTACTAGAGATTGAAACTCC | Mutagensis |
| KARSF291VRv | GGAGTTTCAATCTCTAGTACTCCCAGCTCATCTAAGAAACTTC | Mutagensis |
| KARSI346TFw | CATCGACCGGGTTTATGAAACTGGACGCCAGTTCCGGAATGAG | Mutagensis |
| KARSI346TRv | CTCATTCCGGAACTGGCGTCCAGTTTCATAAACCCGGTCGATG | Mutagensis |
| KARSP499LFw | GATCACCCACAGATAATGAGTCTTTTGGCTAAATGGCACCGCTC | Mutagensis |
| KARSP499LRv | GAGCGGTGCCATTTAGCCAAAAGACTCATTATCTGTGGGTGATC | Mutagensis |
| KARSP533RFw | CAATGCGTATACTGAGCTGAATGATCGCATGCGGCAGCGGCAGC | Mutagensis |
| KARSP533RRv | GCTGCCGCTGCCGCATGCGATCATTCAGCTCAGTATACGCATTG | Mutagensis |
| KARSF585CFw | GCATTGATCGAGTCGCCATGTGCCTCACGGACTCCAACAAC | Mutagensis |
| KARSF585CRv | GTTGTTGGAGTCCGTGAGGCACATGGCGACTCGATCAATGC | Mutagensis |
| KARSN591IFw | GTTTCTCACGGACTCCAATATTATCAAGGAAGTACTTCTGTTTCC | Mutagensis |
| KARSN591IRv | GGAAACAGAAGTACTTCCTTGATAATATTGGAGTCCGTGAGAAAC | Mutagensis |
| CYC1SFw | CACACAAACACAAATACACACAC | Sequencing |
| CYC1SRv | GCGTGAATGTAAGCGTGAC | Sequencing |

**Supplementary Table 2.** Mitochondrial score, of all the patients reported in the manuscript with *KARS*-related disorder.

| **Case** | **Variants** | **Mitochondrial score** |
| --- | --- | --- |
| 1 | p.Phe585Cys/p.Gln75Ser fs*2 | 6 |
| 2 | p.Pro499Leu/p.Phe291Val | 8 |
| 3 | p.Pro533Arg/p.Ala57Pro | 4 |
| 4 | p.Pro533Arg/p.Ala57Pro | 4 |
| 5 | p.Arg205Cys/p.Arg205Cys | 7 |
| 6 | p.Arg205Cys/p.Arg205Cys | 6 |
| 7 | p.Ile346Thr/p.Ile346Thr | 6 |
| 8 | p.Arg108Cys/p.Ala2Val | 7 |
| 9 | p.Arg348Cys/p.His402Tyr | 8 |

**Supplementary Table 3.** Bioinformatic predictions and ACMG classification of *KARS1* variants.

| **Amino acid change in *KARS1*** | **GnomAD** | **SIFT pred** | **Polyphen2 HDIV score** | **MutationTaster score** | **PROVEAN pred** | **M-CAP pred** | **CADD phred** | **phastCons100way vertebrate** | **ACMG classification** |
| --- | --- | --- | --- | --- | --- | --- | --- | --- | --- |
| NM_005548:exon1:c.5C>T:p.(Ala2Val) | 8,18E-03 | D | 0.999 | 0.796 | N | D | 12.81 | 1.000 | PM3, PP3, PP4, BS3 |
| NM_005548:exon2:c.85G>C:p.(Ala29Pro)  NM_001130089:exon3:c.169G>C:p.(Ala57Pro) | 2,03E-02 | D | 1.0 | 1 | D | D | 32 | 1.000 | PS1, PS3, PM2, PP1 |
| NM_005548:exon2:c.139del:p.(Gln47Serfs*1)  NM_001130089:exon3:c.223delC:p.(Gln75Serfs*1) |  |  |  |  |  |  |  |  | PVS1, PM2, PM3 |
| NM_005548:exon3:c.238C>T:p.(Arg80Cys)  NM_001130089:exon4:c.322C>T:p.(Arg108Cys) | 8,13E-03 | D | 0.998 | 1 | D | D | 24.0 | 1.000 | PS3, PM2, PM3, PM5 |
| NM_005548:exon5:c.529C>T:p.(Arg177Cys)  NM_001130089:exon6:c.613C>T:p.(Arg205Cys) | . | D | 0.791 | 1 | D | D | 27.0 | 1.000 | PS3, PM2, PP1, PP2, PP3, PP4 |
| NM_005548:exon6:c.787T>G:p.(Phe263Val)  NM_001130089:exon7:c.871T>G:p.(Phe291Val) | 4,06E-03 | D | 1.0 | 1 | D | D | 30 | 1.000 | PS1, PS3, PM2, PM3, PP2, PP3, PP4 |
| NM_005548:exon8:c.953T>C:p.(Ile318Thr)  NM_001130089:exon9:c.1037T>C:p.(Ile346Thr) | . | D | 0.997 | 1 | D | D | 28.0 | 1.000 | PS1, PS3, PM2, PM3, PP2, PP3, PP4 |
| NM_005548:exon8:c.958C>T:p.(Arg320Cys)  NM_001130089:exon9:c.1042C>T:p.(Arg348Cys) | . | D | 0.99 | 1 | D | D | 34 | 1.000 | PS3, PM2, PM3, PM5, PP2, PP3, PP4 |
| NM_005548:exon9:c.1120C>T:p.(His374Tyr)  NM_001130089:exon10:c.1204C>T:p.(His402Tyr) | . | D | 0.17 | 1 | D | D | 28.1 | 1.000 | PS3, PM2, PM3, PP2, PP3, PP4 |
| NM_005548:exon11:c.1412C>T:p.(Pro471Leu)  NM_001130089:exon12:c.1496C>T:p.(Pro499Leu) | . | D | 1.0 | 1 | D | D | 34 | 1.000 | PS3, PM2, PM3, PP2, PP3, PP4 |
| NM_005548:exon12:c.1514C>G:p.(Pro505Arg)  NM_001130089:exon13:c.1598C>G:p.(Pro533Arg) | . | D | 1.0 | 1 | D | D | 28.4 | 1.000 | PS3, PM2, PM3, PM5, PP1, PP2, PP3, PP4 |
| NM_005548:exon13:c.1670T>G:p.(phe557Cys)  NM_001130089:exon14:c.1754T>G:p.(Phe585Cys) | . | D | 0.999 | 1 | D | D | 28.5 | 1.000 | PS3, PM2, PM3, PP2, PP3, PP4 |
